# GLS1 is a Protective Factor in Patients with Ovarian Clear Cell Carcinoma and its Expression Does Not Correlate with ARID1A-mutated Tumors

**DOI:** 10.1158/2767-9764.CRC-22-0122

**Published:** 2022-08-10

**Authors:** Valentino Clemente, Asumi Hoshino, Mihir Shetty, Andrew Nelson, Britt K. Erickson, Ruth Baker, Nathan Rubin, Mahmoud Khalifa, S. John Weroha, Emil Lou, Martina Bazzaro

**Affiliations:** 1 Masonic Cancer Center and Department of Obstetrics, Gynecology and Women's Health, University of Minnesota, Minneapolis, Minnesota.; 2 Department of Laboratory Medicine and Pathology, University of Minnesota, Minneapolis, Minnesota.; 3 Biostatistics Core, Masonic Cancer Center, University of Minnesota, Minneapolis, Minnesota.; 4 Departments of Oncology and Molecular Pharmacology, Mayo Clinic, Rochester, Minnesota.; 5 Division of Hematology, Oncology, and Transplantation, Department of Medicine, University of Minnesota, Minneapolis, Minnesota.

## Abstract

Targeting glutamine metabolism has emerged as a novel therapeutic strategy for several human cancers, including ovarian cancer. The primary target of this approach is the kidney isoform of glutaminase, glutaminase 1 (GLS1), a key enzyme in glutamine metabolism that is overexpressed in several human cancers. A first-in-class inhibitor of GLS1, called CB839 (Telaglenastat), has been investigated in several clinical trials, with promising results. The first clinical trial of CB839 in platinum-resistant patients with ovarian cancer is forthcoming. *ARID1A*-mutated ovarian clear cell carcinoma (OCCC) is a relatively indolent and chemoresistant ovarian cancer histotype. In OCCC-derived cells ARID1A simultaneously drives GLS1 expression and metabolism reprograming. In ARID1A-mutated OCCC-derived mouse models, loss of ARID1A corresponds to GLS1 upregulation and increases sensitivity to GLS1 inhibition. Thus, targeting of GLS1 with CB839 has been suggested as a targeted approach for patients with OCCC with tumors harboring *ARID1A* mutations. Here, we investigated whether GLS1 is differentially expressed between patients with OCCC whose tumors are ARID1A positive and patients whose tumors are ARID1A negative. In clinical specimens of OCCC, we found that GLS1 overexpression was not correlated with ARID1A loss. In addition, GLS1 overexpression was associated with better clinical outcomes. Our findings have implications for human trials using experimental therapeutics targeting GLS1.

**Significance:**

GLS1 differential expression in patients with OCCC with or without ARID1A mutations is significant because a clinical trial with a GLS1 inhibitor is forthcoming. Tumors without ARID1A have low levels of GLS1 and GLS1 expression is associated to better outcome. Thus, blockade of GLS1 could be counterproductive for patients with OCCC.

## Introduction

Ovarian clear cell carcinoma (OCCC) is an indolent form of ovarian cancer associated with a poor prognosis. The principal reason for such a dismal prognosis is resistance to standard-of-care chemotherapy used in general for ovarian carcinomas, including taxane- and platinum-based agents (1). OCCC is characterized by a specific subset of genetic mutations, the most frequent one being inactivating mutations (protein loss) of *ARID1A,* which is found in approximately 50% of patients (1–5). ARID1A is a member of the SWI/SNF (SWIft/Sucrose Non-Fermentable) complex of chromatin remodelers and is considered a tumor suppressor (5). Mutations in genes coding for members of the SWI/SNF complex are found in approximately 20% of all human cancers (6). Patients with OCCC carrying *ARID1A* mutations, including patients that are diagnosed at early stages, have worse prognoses than patients without *ARID1A* mutations (5, 7–15). Thus, extensive literature indicates that patients carrying these mutations may be less responsive to treatment. Hence, there is a notable urgency to discover new and more effective treatments based on the molecular dependencies of *ARID1A*-mutated OCCC.

We and others have contributed to the understanding of how the SWI/SNF remodeling complex controls the energetic metabolism of mammalian cells, including the mitochondrial metabolism of cancer cells. In fact, we have recently shown that ARID1A loss is associated with higher dependency upon mitochondrial respiration and selective sensitivity to its inhibition, both *in vitro* and in a preclinical model of *ARID1A*-mutated OCCC (16). This result is consistent with a previous study showing that OCCC-derived cells have higher mitochondrial respiration as compared to ovarian cancer cell lines derived from other histotypes (17). It is also consistent with another study showing that ARID1A loss leads to selective sensitivity to ROS- inducing agents in OCCC cells (18). Interestingly, it has been recently shown that in ARID1A knock-out cells, increased glutamine metabolism and expression of GLS1, the key regulating enzyme in glutamine metabolism, are responsible for fueling mitochondrial respiration (19). Thus, inhibition of GLS1 with the novel inhibitor CB839 (Telaglenastat) has been proposed as a novel strategy for the treatment of *ARID1A*-mutated OCCC (19). This is conceptually supported by findings from our team (16) and others (20–24) showing that in OCCC, ARID1A loss is followed by increased expression levels of c-Myc, which is known to be an important regulator of GLS1 expression (25–47). In this scenario and given that the mitochondrial pathways involve a number or proteins organized in complexes and super complexes, GLS1 would represent an ideal marker and molecular target for *ARID1A*-mutated tumors as it represents the “bottleneck” for mitochondrial glutamine metabolism. Hence, there is strong enthusiasm and scientific rationale for the launch of a forthcoming clinical trial utilizing CB839 in ovarian cancer including OCCC (48).

In this study, we addressed the question of whether GLS1 is differentially expressed between patients with OCCC whose tumors are *ARID1A* wild-type and ARID1A-positive versus patients whose tumors are *ARID1A*-mutated and ARID1A negative. This question is relevant because it would provide a predictive biomarker for OCCC being treated with CB839. We found that in clinical specimens of OCCC, GLS1 overexpression was not correlated to ARID1A loss and, on the contrary, was associated with better clinical outcome. This result suggests that in OCCC GLS1 may be a protective factor and that caution should be taken when considering the use of CB839 to treat patients with OCCC and especially those with *ARID1A* mutations.

## Materials and Methods

### Antibodies

Anti-ARID1A (HPA005456, Sigma-Aldrich) and anti-GLS1 (ab156876, clone EP7212, Abcam).

### Human Subjects

Archival tissues were used with approval from the Institutional Review Board (IRB) of the University of Minnesota (STUDY00006529). Demographic information and patient characteristics are reported in Supplementary Table S1. For the second cohort of OCCC specimens, tissue collection and use were approved by the Mayo Clinic IRB (09–008768). All tissues were obtained with the written informed consent of the patients and the studies were conducted in accordance with the recognize ethical guidelines of the Belmont Report.

### Immunohistochemistry

Representative cores from the formalin fixed paraffin embedded (FFPE) blocks of archival tissues were selected and arranged in (two) tissue microarrays (TMA) containing a total of sixty clinical specimens. Fifty-four specimens had enough quality tissue for both ARID1A and GLS1 staining. Thus, the correlation between GLS1 and ARID1A expression was calculated based on 54 (37 + 17) of these specimens. Clinical information was available for 55 ARID1A-stained specimens (Supplementary Table S1) and 54 GLS1-stained specimens (Supplementary Table S2). For the second cohort of OCCC tissues, TMAs were not available so individual tissue blocks were used for each patient tumor. Five micron–thick, formalin-fixed, paraffin-embedded (FFPE) TMA sections were deparaffinized and rehydrated by sequential washing with xylene, 100% ethanol, 95% ethanol, 80% ethanol, and PBS. Antigen retrieval was then carried out with 1× Reveal Decloaker (Biocare Medical) in a vegetable steamer for 30 minutes at 100°C, before blocking the slides with Background Sniper (BS966H, Biocare Medical, Pacheco, CA, USA) for 13 min at room temperature. After washing with PBS, sections were incubated with anti-ARID1A (1:250 dilution) or ant-GLS1 (1:100 dilution) antibodies overnight at 4°C. After washing twice with PBS, the sections were incubated with Biotin-SP–conjugated AffiniPure Goat Anti-Rabbit IgG (111–065–003, Jackson ImmunoResearch Laboratories) at a dilution of 1:200 for 30 minutes at room temperature followed by incubation with horseradish peroxidase streptavidin at a dilution of 1:125 (405210, BioLegend) for 30 minutes at room temperature. After the staining was developed with 3,3′-diaminobenzidine (926506, BioLegend) for 3 minutes, slides were counterstained with Harris’ hematoxylin. Immunostained slides were reviewed by a panel of two investigators blinded to the clinical outcome of the corresponding patients. ARID1A immunoreactivity was scored using an immunoreactive score (IRS), and a cutoff of <5.5 was considered predictive of mutation, as previously described (49). This IHC staining approach is the most widely used for *ARID1A* mutation screening and as a method that has been shown to reliably predict *ARID1A* mutations with 100% sensitivity and specificity. GLS1 immunoreactivity was scored using an H-score: staining intensities (0 = no staining, 1 = weak, yellow staining, 2 = yellow/brown, 3 = brown) were multiplied by their respective percentages of cells stained (final range: 1 to 300). Brightfield images were acquired with a Zeiss Axio Scan.Z1 system (Zeiss) at 40× magnification.

### Exome Sequencing

Somatic DNA was extracted from FFPE tissue samples. Next-generation sequencing (NGS) libraries were prepared with 50–250 ng of purified DNA following the Illumina NGS library preparation procedure and enriched by PredicineCARE NGS panel (Predicine) using a hybrid capture method and deeply sequenced by Illumina pair-end sequencing. Raw sequencing data (BCL files) were fed through DeepSea, a Predicine proprietary NGS analysis pipeline. Paired-end reads were first merged as single-strand fragments, then the consensus bam file was built by merging fragments with the same start and end mapping locations. Raw variants were called from mismatches with the reference genome in the consensus bam file and were then filtered on the basis of variant background (defined by normal plasma samples and historical data), repeat regions, and other quality metrics. Variants with mutation allele frequency (MAF) ≥ 5% and hotspot variants with MAF down to 2% were reported.

### Western Blot Analysis

Total cellular protein (10–30 μg) from each sample was separated by SDS-PAGE, transferred to polyvinylidene fluoride (PVDF) membranes and subjected to Western blot analysis using the specified antibodies, as previously shown (16). Amido black staining was used to confirm equal protein loading. Lysates from control and ARID1A knock-out (KO) cells (19) were a generous gift from Dr. Rugang Zang (Wistar Institute).

### Statistical Analysis

Patient demographic and clinical measures were summarized and compared between ARID1A and GLS1 categorical groups using either one-way ANOVA or 2 sample *t* tests for continuous measures and Fisher exact tests for categorical measures. In this analysis ARID1A was treated as a binary variable using the previously mentioned cut-off of 5.5, while GLS1 categorical groups were created based on low (<100), moderate (<200, ≥100) and high (≥200) expression levels. Survival curves were generated using the Kaplan–Meier method. Univariate Cox regression models were used to determine the statistical significance of ARID1A and GLS1 on progression-free survival (PFS), cancer-specific survival (CSS), and overall survival (OS). After identifying possible confounders, models were also generated to adjust for factors such as stage and endometriosis (50). In the survival analyses, GLS1 was treated as a continuous variable. R (Version 3.4.1, The R Foundation for Statistical Computing) was used for demographic and survival analyses, while graphs were obtained using GraphPad Prism (version 8.4.3). ARID1A status (< or >5.5) and GLS1 H-score distributions were analyzed using GraphPad Prism. *P* values of <0.05 were considered statistically significant.

### Data Availability

Whole-exome sequencing was performed by a CLIA-certified laboratory (Predicine) and raw data were not available at the time of publication due to Predicine's legal terms and conditions. Only the mutation calling data was provided by Predicine. All available data will be shared upon reasonable request from the corresponding author.

## Results

### ARID1A Loss Negatively Correlates with the Prognosis of Patients with OCCC

**FIGURE 1:**
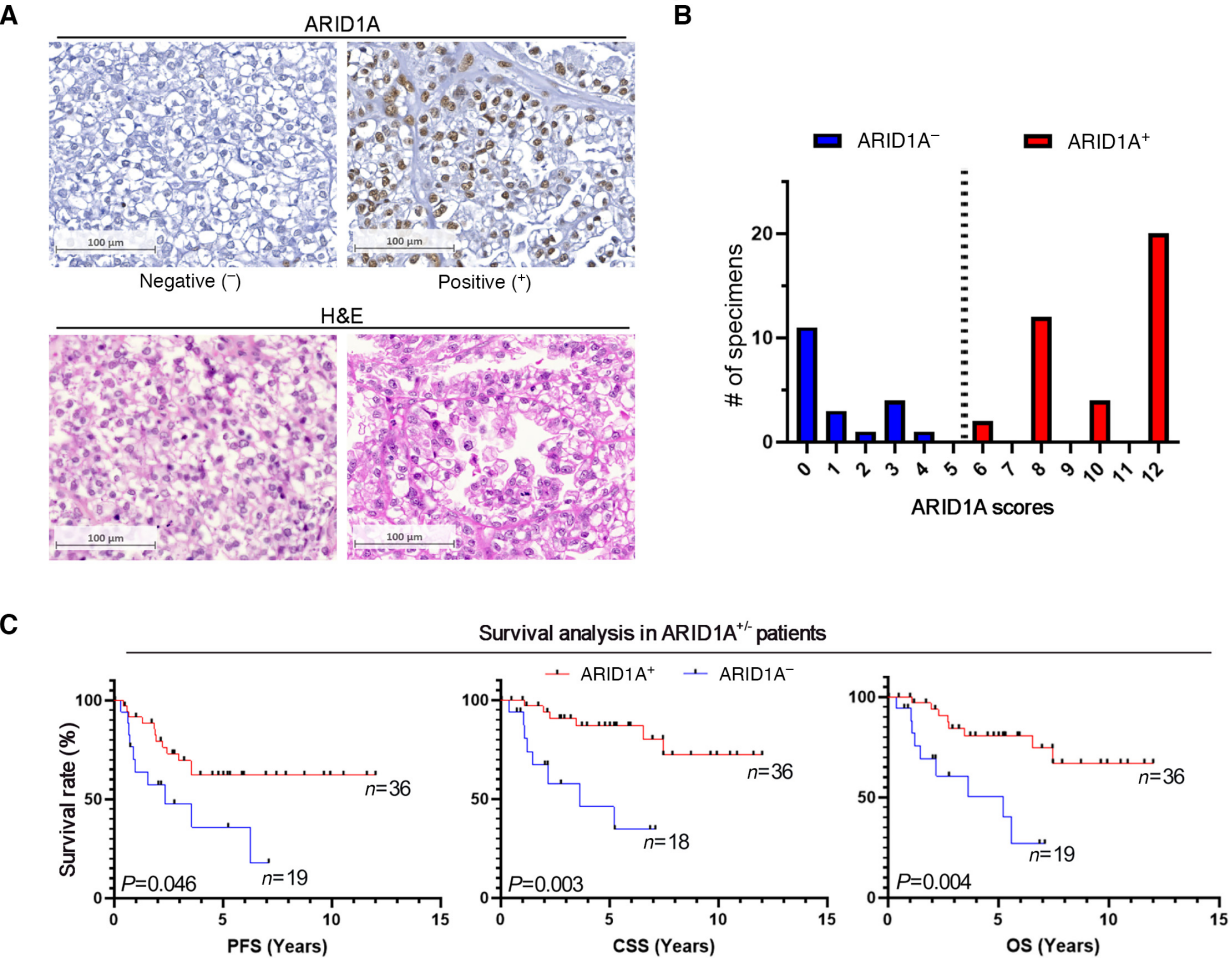
ARID1A loss negatively correlates with the prognosis of 0.24–1.49OCCC. **A,** Top, representative images of ARID1A-negative or -positive OCCC clinical specimens and their respective Frequency distribution of the immunoreactivity scores for ARID1A; the dotted line represents the cutoff used to discriminate between ARID1A positive (+) and ARID1A negative (−) patients. **C,** Survival curves of ARID1A^+^*vs.* ARID1A^−^ patients expressed in years. From the left to the right: PFS [36 vs. 19; *P =* 0.046; HR = 0.43; 95% confidence interval (CI), 0.19–0.98], cancer-specific survival, CSS (36 vs. 18; *P =* 0.003; HR = 0.18; 95% CI, 0.06–0.55), overall survival (OS; 36 vs. 19; *P =* 0.004; HR = 0.23, 95% CI, 0.09–0.63).

Studies conducted in both OCCC and in endometrial carcinoma have shown that *ARID1A* mutations are found in 50 and 30% of patients, respectively (2, 5, 9, 15, 49, 51) and that patients with tumors carrying *ARID1A* mutations have worse prognosis as compared with patients who do not. This finding is true even for patients diagnosed with early-stage disease (7–15). For these reasons, ARID1A mutations have been proposed as a co-factor for worse prognoses. Thus, we first validated our OCCC clinical cohort by performing ARID1A IHC staining (Fig. 1A) to correlate ARID1A expression levels with clinical outcome. Patient demographics and characteristics are listed in Supplementary Table S1. Importantly, IHC is the most commonly used technique to evaluate ARID1A expression levels (5). This method has been shown to reliably predict *ARID1A* mutations in 76% of cases when using a no immunoreactivity cut-off (2) and with 100% sensitivity and specificity when using an IRS <5.5 cut-off (49). This highly reliable method for determining ARID1A status via IHC was used in our study. As shown in Fig. 1B, we found a distribution of the scores similar to what has been previously shown (49) with 19 ARID1A-negative samples out of 55 (34.5%). Specifically, the “0” on the “*y*” axis of the distribution score indicates that none of the specimens on the array was assigned the intensity scores of 5, 7, 9 and 11. The “0” on the “*x*” axis of the distribution score indicates that 11 specimens had an ARID1A intensity of 0 which is consistent with ARID1A mutational status. This result is consistent with the prevalence of *ARID1A* mutations, which is normally in the range between 30% and 70% of OCCC cases (2, 5, 15, 49, 51). Notably, we found that loss of ARID1A occurred more frequently in patients with endometriosis found during pathologic diagnosis, and also had a tendency to present at lower stages (Supplementary Table S1). This is also consistent with the fact that ARID1A mutations are frequently associated with endometriosis (1, 2, 52–57).

**FIGURE 2:**
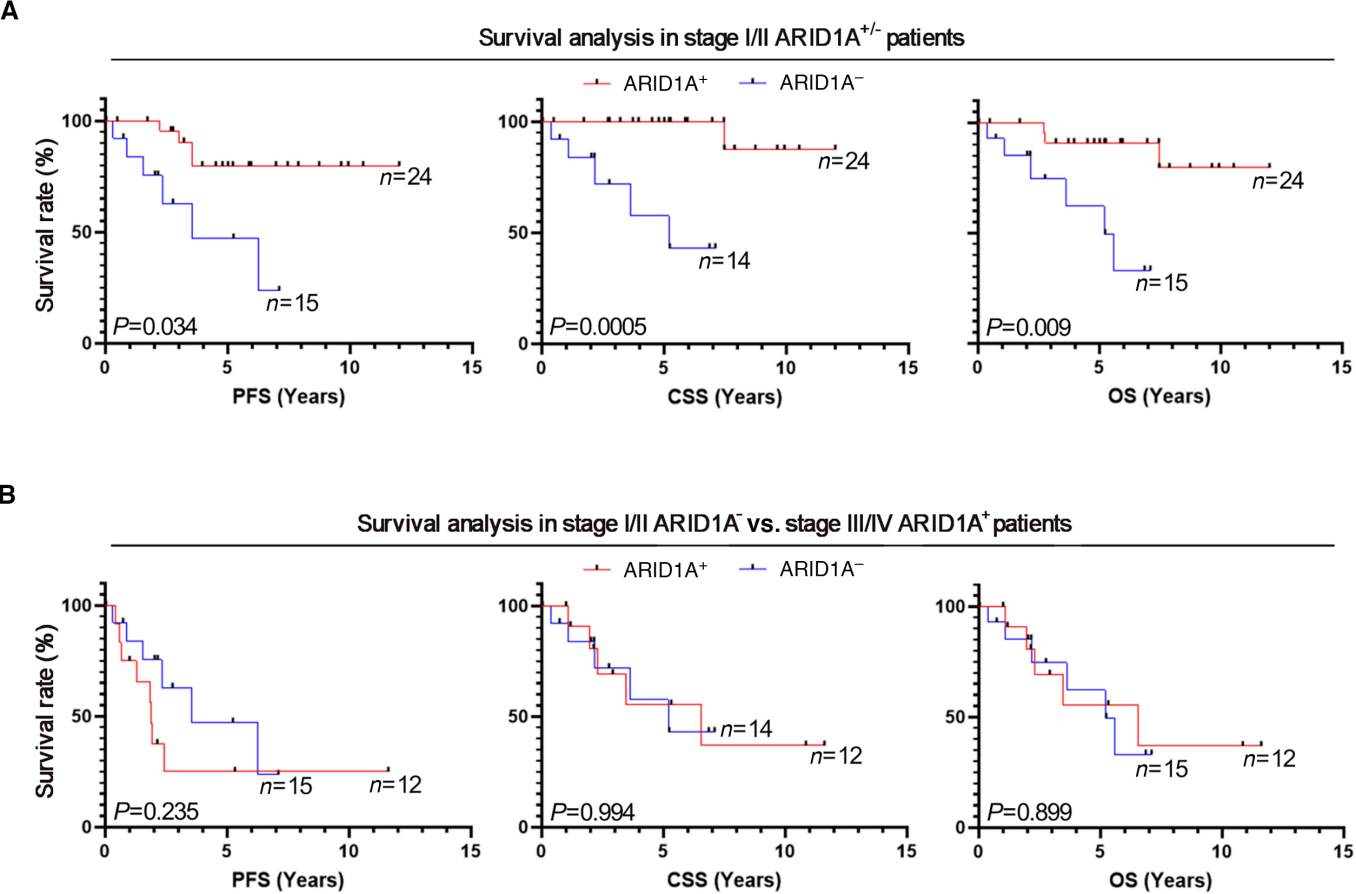
Stage I/II ARID1A negative patients have the same prognoses as stage III/IV ARID1A-positive patients. **A,** Survival curves of ARID1A^+^ (red) vs. ARID1A^−^ (blue) stage I/II patients expressed in years. From the left to the right: PFS (24 vs. 15; *P =* 0.034; HR = 0.29; 95% CI, 0.09–0.91), CSS (24 vs. 14; *P =* 0.0005; HR = 17.27; 95% CI, 2.538–117.6 – Mantel-Cox's logrank test), OS (24 vs. 15; *P =* 0.009; HR = 0.12; 95% CI, 0.02–0.59). **B,** Survival curves of stage III/IV ARID1A^+^ (red) vs. stage I/II ARID1A^−^ (blue) patients expressed in years. From the left to the right: PFS (12 vs. 15; *P =* 0.235; HR, 0.52; 95 CI, 0.18–1.52), CSS (12 vs. 14; *P =* 0.994; HR, 1.01; 95% CI, 0.29–3.49), OS (12 vs. 15; *P =* 0.899; HR, 1.08; 95% CI, 0.33–3.56).

We then sought to determine the correlation between ARID1A expression and clinical outcome. As shown in Fig. 1C, we found a significant and strong detrimental effect for ARID1A loss on PFS (*P* = 0.046), CSS (*P* = 0.003), and OS (*P* = 0.004). Importantly, the correlation between ARID1A loss and worse outcome remained significant even after adjusting for stage and endometriosis in multivariate analysis (Table 1; PFS, *P* = 0.004; CSS, *P* < 0.001; OS, *P* < 0.001). Classification of early stages (I+II) versus late stage (III–IV) is common practice both clinically and in retrospective and prospective studies involving patients with ovarian cancer because these two groups typically have very different outcomes. In fact, consistent with other studies (9) an even larger effect of ARID1A loss on survival was found in stage I/II patients (Fig. 2A; PFS, *P* = 0.034; CSS, *P* < 0.001; OS, *P* = 0.009), with early stage ARID1A-negative patients having a prognosis similar to that of III/IV ARID1A-positive patients (Fig. 2B; PFS, *P* = 0.235; CSS, *P* = 0.994; OS, *P* = 0.899). Taken together, these results demonstrate that our cohort can reliably be used to investigate the correlation between ARID1A status and GLS1.

**TABLE 1:** Multivariate analysis for ARID1A.

	PFS		CSS		OS	
Characteristics	HR (95% CI)	*P*	HR (95% CI)	*P*	HR (95% CI)	*P*
ARID1A						
Negative						
Positive	0.21 (0.07, 0.60)	**0.004**	0.04 (0.01, 0.21)	**<0.001**	0.08 (0.02, 0.30)	**<0.001**
Endometriosis						
Yes						
No	1.42 (0.53, 3.81)	0.486	2.54 (0.66, 9.71)	0.173	2.28 (0.74, 7.00)	0.149
Stage						
I/II						
III/IV	7.80 (3.05, 19.9)	**<0.001**	14.4 (3.42, 60.9)	**<0.001**	8.05 (2.36, 27.5)	**<0.001**

### GLS1 Expression is Negatively Correlated With ARID1A Loss in OCCC

**FIGURE 3:**
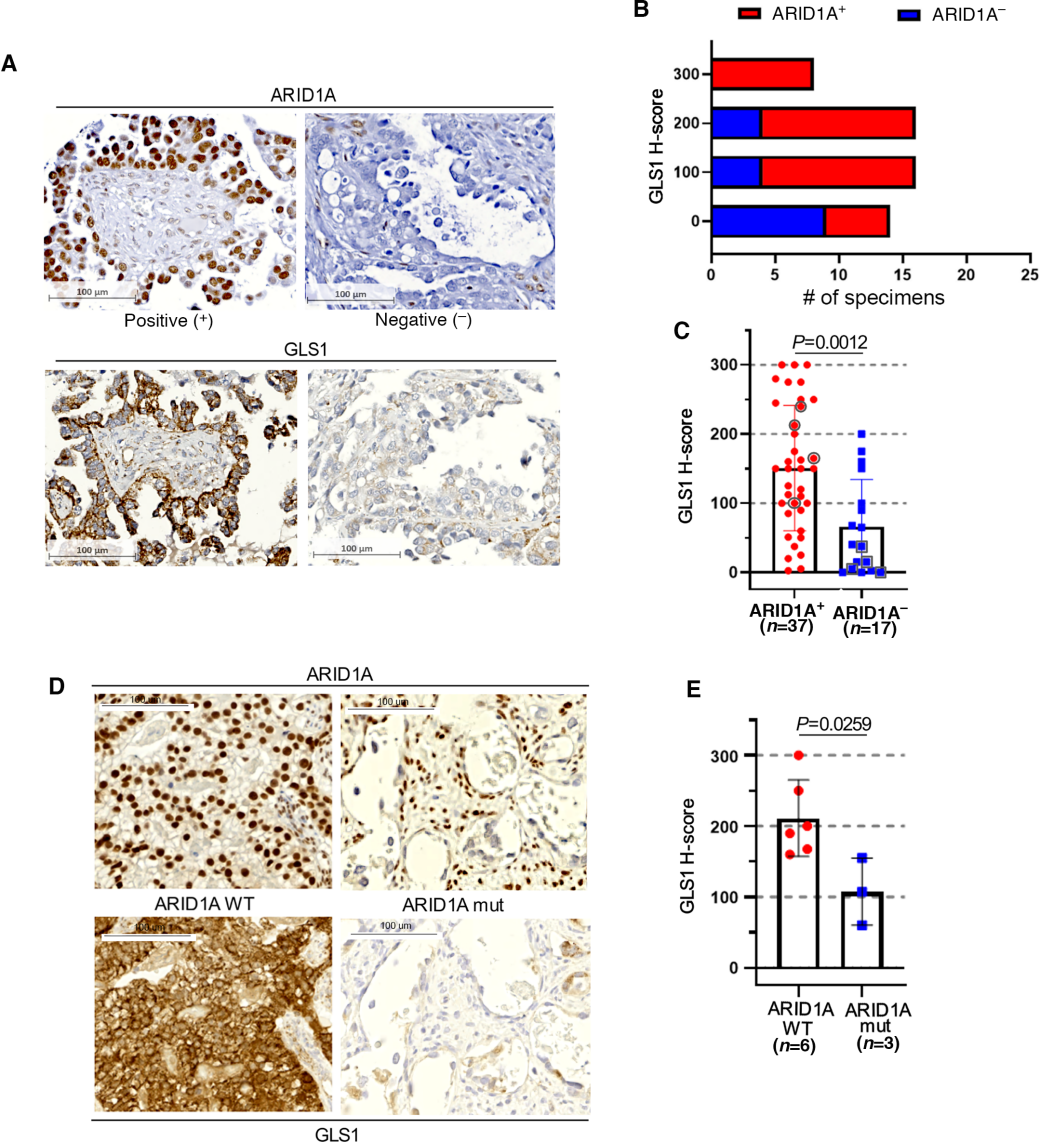
GLS1 expression is negatively correlated with ARID1A loss in OCCC. **A,** Top, representative images of GLS1 IHC staining in ARID1A-positive and ARID1A-negative OCCC specimens; bottom*,* respective ARID1A immunostainings. **B,** Frequency distribution of the GLS1 H-scores in ARID1A^+^ and ARID1A^−^ OCCC. **C,** Expression levels of GLS1 in ARID1A^+^ and ARID1A^−^ OCCC (ARID1A^+^ vs. ARID1A^−^: *P =* 0.0012). *n* = number of clinical specimens per group. The circled dots represent the cases for which an additional Western blot analysis has been performed. **D,** Representative images of GLS1 IHC staining in ARID1A wild-type (WT) and ARID1A-mutated (mut.) OCCC specimens. **E,** Expression levels of GLS1 in ARID1A wild-type (WT) and ARID1A-mutated (mut) OCCC. *n* = number of clinical specimens per group.

Recent reports have shown that GLS1 is overexpressed in both OCCC and high grade-serous carcinoma (HGSC) of the ovaries, compared with cells derived from normal surface epithelium of the ovaries and fallopian tubes (25). Furthermore, in HGSC, GLS1 is overexpressed in clinical tumor specimens from patients who are chemoresistant versus patients whose tumors are responsive to chemotherapy (25). Thus, GLS1 has been proposed as a rational molecular target for chemoresistant ovarian cancer. Here, we sought to assess whether GLS1 is differentially expressed between patients with OCCC whose tumors are ARID1A positive and patients whose tumors are ARID1A negative. We found that GLS1 is not overexpressed in clinical specimens of OCCC that are negative for ARID1A (Fig. 3A). On the contrary, we found that ARID1A-positive tumors have higher levels of GLS1 as compared with patients who are ARID1A negative (Fig. 3; *P* = 0.001). Evaluation of ARID1A expression via IHC has been shown to reliably predict *ARID1A* mutations (49). To experimentally confirm this, we turned to a second OCCCs representative cohort for which next-generation exosome sequencing was previously performed in our laboratories. We used this cohort to determine ARID1A expression levels via IHC as done for the first cohort. As shown in Table 2, we found that consistent with the previously published work (49), patients whose tumors carried *ARID1A* mutations had a dramatically lower ARID1A expression levels (H-score) as compared with patients who did not carry *ARID1A* mutations. The fact that in the OCCC cohort for which we had performed next-generation exosome sequencing, patients whose tumors carried *ARID1A* mutations had a dramatically lower ARID1A expression levels (H-score) as compared to patients who did not carry *ARID1A* mutations further speaks for the reliability of IHC staining to determine ARID1A mutational status.

**TABLE 2:** ARID1A's H-score and exome sequencing in OCCCs specimens.

Specimen ID	H-score	Variant classification	Clinical significance	Exon number
738	<40	Nonsense mutation	Likely pathogenic	10
250	<40	Nonsense mutation	Pathogenic	20
982	<40	Nonsense mutation	Pathogenic	16
441	>200			
847	>200			
936	>200			
951	>200			
800	>200			
985	>200			

**FIGURE 4:**
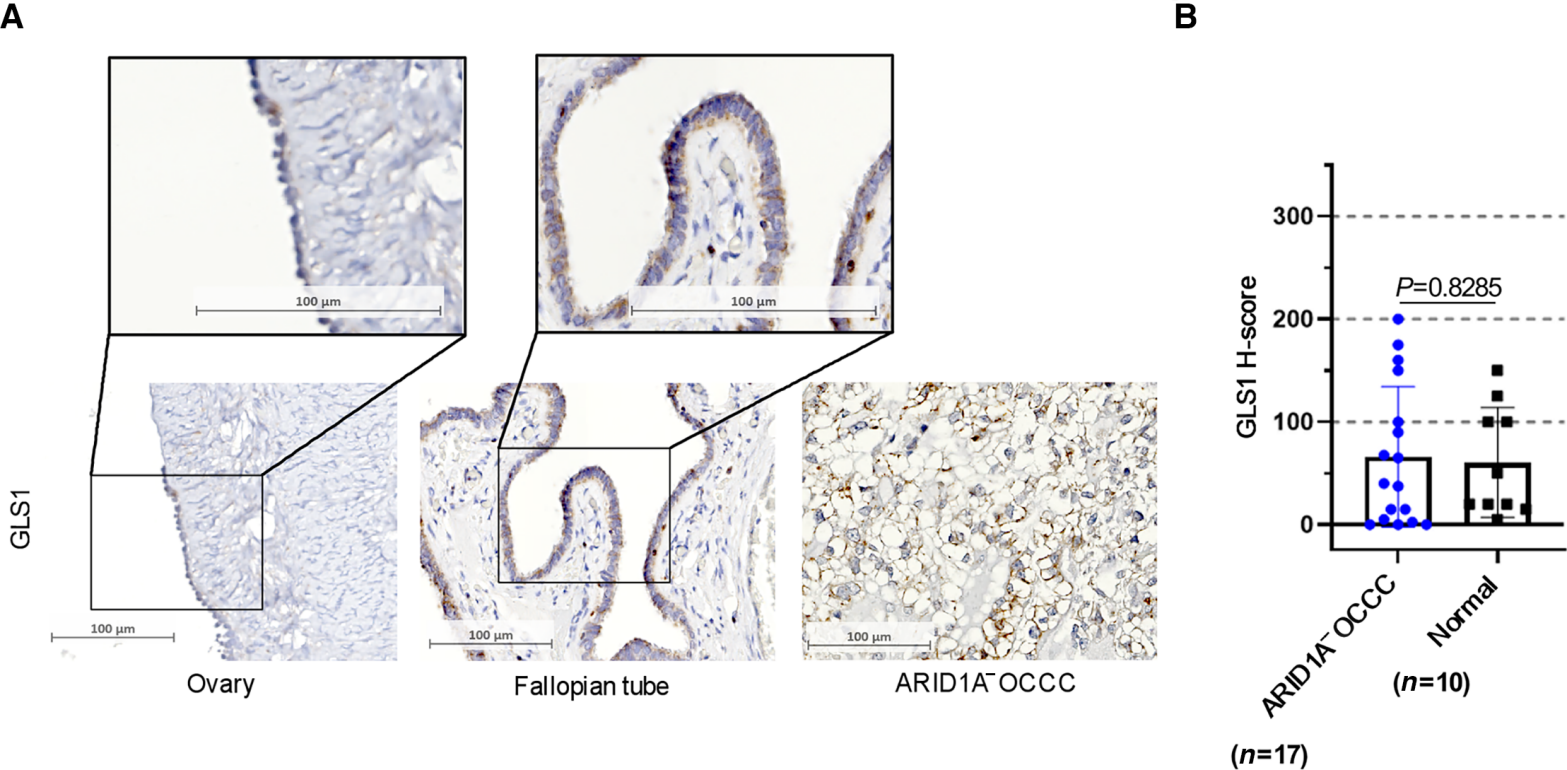
GLS1 expression in ARID1A-negative OCCC is similar to the one of normal tissues. **A,** Bottom left and its inset, representative image of GLS1 staining in normal ovary. Bottom center and its inset, representative image of GLS1 staining in normal Fallopian tube. Bottom right, representative image of GLS1 staining in ARID1A-negative (−) OCCC. **B,** Expression levels of GLS1 in ARID1A-negative OCCC and in normal tissues. (ARID1A- *vs.* normal: *P =* 0.8285). *n* = number of clinical specimens per group.

Next, we evaluated the expression levels of GLS1 in this second representative cohort. We found that, consistent with our findings in the first cohort we analyzed, ARID1A wild type (WT) tumors have higher levels of GLS1 as compared with ARID1A-mutated tumors (*P* = 0.0259). This finding is novel and unanticipated. We then compared GLS1 expression levels between ARID1A-negative OCCC and normal surface epithelia from ovaries and fallopian tubes. We found that GLS1 expression in normal epithelia is similar to expression levels detected in ARID1A-negative OCCC (Fig. 4 and Supplementary Fig. S1A; *P* = 0.828). Moreover, GLS1 levels were not correlated with other clinicopathologic parameters, suggesting that the ARID1A status was the only determinant of GLS1 levels in our cohort (Supplementary Table S2). Furthermore, we also validated our findings biochemically via Western blot analysis. Specifically, we first went back to an *in vitro* system and assessed GLS1 and ARID1A levels in ARID1A KO RMG1 cells. As shown in Supplementary Fig. S1b, while ARID1A immunoreactivity was abolished, GLS1 was strongly overexpressed in ARID1A KO vs. ARID1A WT cells, consistently with what had previously been shown (19). This also indicates that the Ab we used is selective and specific for ARID1A.

Next, we selected a representative cohort of 4 ARID1A-positive and 4 ARID1A-negative OCCC clinical specimens (as assessed via IHC) for which we had paired frozen specimens available (Fig. 3C). As shown by Western blot in Supplementary Fig. S1C and S1D, while ARID1A was undetectable in the ARID1A-negative specimens, GLS1 was overexpressed in the ARID1A-positive group, thus confirming our results. Furthermore, two out of four ARID1A positive specimens had a strong expression of the KGA isoform of GLS1, while none of the ARID1A-negative specimens had detectable levels of it.

### GLS1 Overexpression May be a Protective Factor in OCCC

**FIGURE 5:**
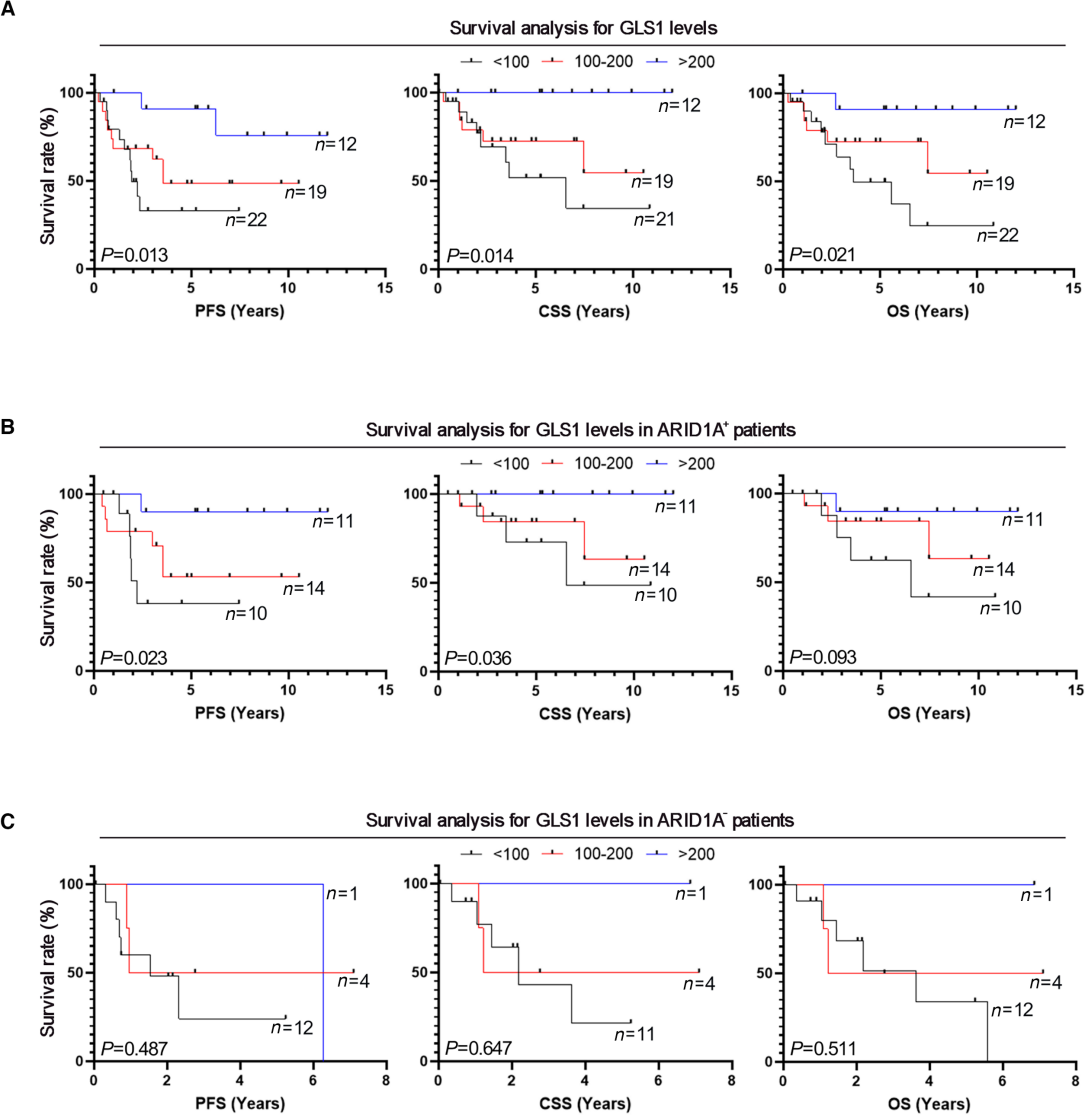
GLS1 overexpression may be a protective factor in OCCC. **A,** Kaplan-Meier curves of OCCC patients, divided by high (≥200, blue), intermediate (<200, ≥100, red) and low (<100, black) GLS1 expression levels. From left to right: PFS (12 vs. 19 vs. 22; *P =* 0.013; HR by 10 points increase = 0.93; 95% CI, 0.88–0.99), CSS (12 vs. 19 vs. 21; *P =* 0.014; HR by 10 points increase = 0.91; 95% CI, 0.85–0.98). OS (12 vs. 19 vs. 22; *P =* 0.021; HR by 10 points increase = 0.93; 95% CI, 0.87–0.99). Survival times are expressed in years. **B,** Kaplan–Meier curves of ARID1A^+^ patients, divided by high (≥200, blue), intermediate (<200, ≥100, red) and low (<100, black) GLS1 expression levels. From left to right: PFS (11 vs. 14 vs. 10, *P =* 0.023, HR by 10 points increase = 0.91; 95% CI, 0.84–0.99), CSS (11 vs. 14 vs. 10, *P =* 0.036, HR by 10 points increase = 0.88; 95% CI, 0.79–0.99), OS (11 vs. 14 vs. 10, *P =* 0.093; HR by 10 points increase = 0.93; 95% CI, 0.85–1.01). Survival times are expressed in years. **C,** Kaplan–Meier curves of ARID1A^−^ patients, divided by high (≥200, blue), intermediate (<200, ≥100, red) and low (<100, black) GLS1 expression levels. From left to right: PFS (1 vs. 4 vs. 12, *P =* 0.487; HR by 10 points increase = 0.96; 95% CI, 0.86–1.07), CSS (1 vs. 4 vs. 11; *P =* 0.647; HR by 10 points increase = 0.97; 95% CI, 0.87–1.09), OS (1 vs. 4 vs. 12; *P =* 0.511; HR = 0.97; 95% CI, 0.87–1.07). Survival times are expressed in years.

We next sought to determine the prognostic significance of GLS1 expression levels in our cohort of OCCC clinical specimens. We found that, while the GLS1 expression levels were not correlated to any specific demographic characteristic of the patients, high GLS1 levels were associated with better survival (Fig. 5A; PFS, *P* = 0.013; CSS, *P* = 0.014; OS, *P* = 0.021). The correlation of GLS1 overexpression with better clinical outcome was novel and unanticipated. The absence of correlation with the clinicopathological characteristics was consistent with previous findings in other cancers (58–61). The reasons for that are likely multifactorial and may include the fact that GLS1 regulation occurs at the cellular or tumor microenvironment level (19, 62) rather than being influenced by the macroscopical parameters usually considered in clinical practice.

Next, because we found that loss of ARID1A immunoreactivity acted as a negative prognostic factor in our set of patients, and that this correlated with low levels of GLS1, we included the ARID1A status in multivariate analysis to exclude the hypothesis that it may have acted as a confounder. Both the factors tended to remain significant, but ARID1A did not reach significance for PFS (PFS, *P* = 0.271; CSS, *P* = 0.026; OS, *P* = 0.042), and GLS1 only showed a trend toward improved OS (PFS, *P* = 0.031; CSS, *P* = 0.033; OS, *P* = 0.064; Table 3). Furthermore, when analyzing the effect of GLS1 on the prognosis of the ARID1A+ and ARID1A- subpopulations individually, all the survival curves showed a similar trend to the general population (Fig. 5B and C; ARID1A+: PFS, *P* = 0.023; CSS, *P* = 0.036; OS, *P* = 0.093), but significance was lost in the ARID1A- subgroup (Fig. 4; PFS, *P* = 0.487; CSS, *P* = 0.647; OS, *P* = 0.511), probably due to the very small size of the moderate and high expression groups (*n* = 4 and 1, respectively).

**TABLE 3:** Multivariate analysis for GLS1.

	PFS		CSS		OS	
Characteristics	HR (95% CI)	*P*	HR (95% CI)	*P*	HR (95% CI)	*P*
*ARID1A*						
* Negative*						
* Positive*	0.60 (0.24–1.49)	0.271	0.25 (0.08, 0.85)	**0.026**	0.33 (0.11, 0.96)	**0.042**
*GLS1*	0.94 (0.88–0.99)	**0.031**	0.92 (0.84, 0.99)	**0.033**	0.94 (0.87, 1.00)	0.064

## Discussion

Clinical behavior of ovarian carcinomas is broad, ranging from highly aggressive to more indolent in nature. This behavior notably affects response to the relatively few standard-of-care treatments available for women affected by this form of cancer. Identification and validation of molecular and cellular biomarkers that can be easily tested and that are predictive of response to specific drugs remain the ideal goal of ovarian cancer research. This fact is especially crucial due to the relative dearth of drugs that induce meaningful clinical response, and even more so in the era of emerging molecular targets. Clear cell carcinomas present a particular treatment challenge. Glutaminase 1 (GLS1) is a key enzyme in glutamine metabolism that has been shown to be overexpressed in cancer cell lines and tumor models and to be required for the survival of senescent cells (63). Furthermore, GLS1 is has been shown to be upregulated in cell lines and mouse models of *ARID1A*-mutant OCCC providing the basis for investigation of this target in the clinical trial setting. However, as we demonstrate here in a large tumor dataset, we found that GLS1 is not overexpressed in clinical specimens of OCCC that are negative for ARID1A and that on the contrary, ARID1A-positive tumors have higher levels of GLS1 as compared to patients who are ARID1A negative. In addition, we found that higher levels of GLS1 associate with better survival rates in patients with ARID1A mutations, even as ARID1A loss negatively correlated with the prognosis of these OCCC patients. The reasons for the discrepancy in expression levels of GLS1 with respect to ARID1A in cell lines and mouse models *versus* human clinical specimens may be multiple and multifactorial. The two most plausible reasons could be a) in the necessarily different microenvironment between cancer cells growing in cultures, cancer cells growing in a mouse and cancer cell growing *in situ* in patients’ tumors, and b) in the difference in proliferation rate of cancer cells *in vitro*, in a mouse model and in a patients’ tumors. This is consistent with recent work showing that *in vitro* glutamine metabolism is highly dependent on experimental conditions (64, 65). Furthermore, we found that GLS1 expression negatively correlated with ARID1A loss in OCCC, and its expression in ARID1A-negative OCCC is like its expression in normal, nonmalignant ovarian and fallopian tube tissue from these patients. We detected no difference when comparing patients with early-stage versus late-stage OCCC.

Importantly, our findings have implications for human trials using experimental therapeutics targeting GLS1. The ability to add ARID1A as a correlative predictive biomarker using IHC and/or next-generation sequencing techniques should be strongly considered for current and forthcoming trials to provide prospective validation of our findings. More importantly, to best stratify patients and limit use of targeted drugs that are not likely to be effective in patients and which may cause more harm than benefit, our findings should be examined carefully before launching iterations of this therapeutic strategy.

Noteworthy, while in this study we have focused on ARID1A as it represents the most frequently mutated component of the SWI/SNF complex in OCCC (1–5), other components have been shown to regulate GLS1 expression in this particular disease (19). Furthermore, regulation of GLS1 levels is a multifactorial process, in which other proteins may have a more important role than ARID1A (25). Finally, the use of GLS1 inhibitors in cancer is also supported by other effects than the ones that glutamine starvation itself may have on cancer cells. For instance, inhibition of GLS1 has been shown to induce cellular stress through the glutathione system (66, 67), which has been proposed as a therapeutic strategy for ARID1A mutated OCCC (68).

Considerations for this study include (i) the retrospective approach; as always, prospective evaluation with appropriate pre-powered statistical design is indicated and necessary to confirm these findings, (ii) sample size; we had available total of sixty clinical specimens in our study. This is an appropriate number considering that ovarian cancer is a rare disease and that OCCC represent 5%–10% of all ovarian carcinomas. However, and as always, a bigger cohort would allow for validation and refining of our findings, *c)* IHC to determine ARID1A levels; in the era of genomic profiling, IHC has been proven to be highly reliable to distinguish between *ARID1A* WT and *ARID1A*-mutated tumors (49). Furthermore, using a second OCCC cohort we have confirmed that mutational status of *ARID1A* correlates with dramatical lower ARID1A's H-score. Importantly, ARID1A protein is the one that necessarily sustains the chromatin remodeling function of ARID1A. It should be kept in mind that investigating protein levels may not be fully indicative of enzymatic activity, although an increase in GLS1 levels is always reported together with increased glutamine metabolism, including in ARID1A-mutated OCCC (19). Finally, investigating if other forms of ARID correlate with GLS1 would be interesting as other members could compensate for the loss of ARID1A. However, because “ARID” contains seven subfamilies and 15 members (69) this may not be feasible due to lack of availability of reliable and validated Abs against other ARID members.

## Supplementary Material

Supplementary Figures S1, Tables S1-S2 Figure S1, Table S1, Table S2
