# Supplementary material for: GLS1 is a Protective Factor in Patients with Ovarian Clear Cell Carcinoma and its Expression Does Not Correlate with ARID1A-mutated Tumors
Source: Cancer Res Commun. 2022 Aug 10;2(8):784–94. doi: 10.1158/2767-9764.CRC-22-0122 (PMC9451103; doi:10.1158/2767-9764.CRC-22-0122)
Supplement: Supplementary Figures S1, Tables S1-S2 — Figure S1, Table S1, Table S2 [file crc-22-0122-s01.pdf]

Supplementary figure 1

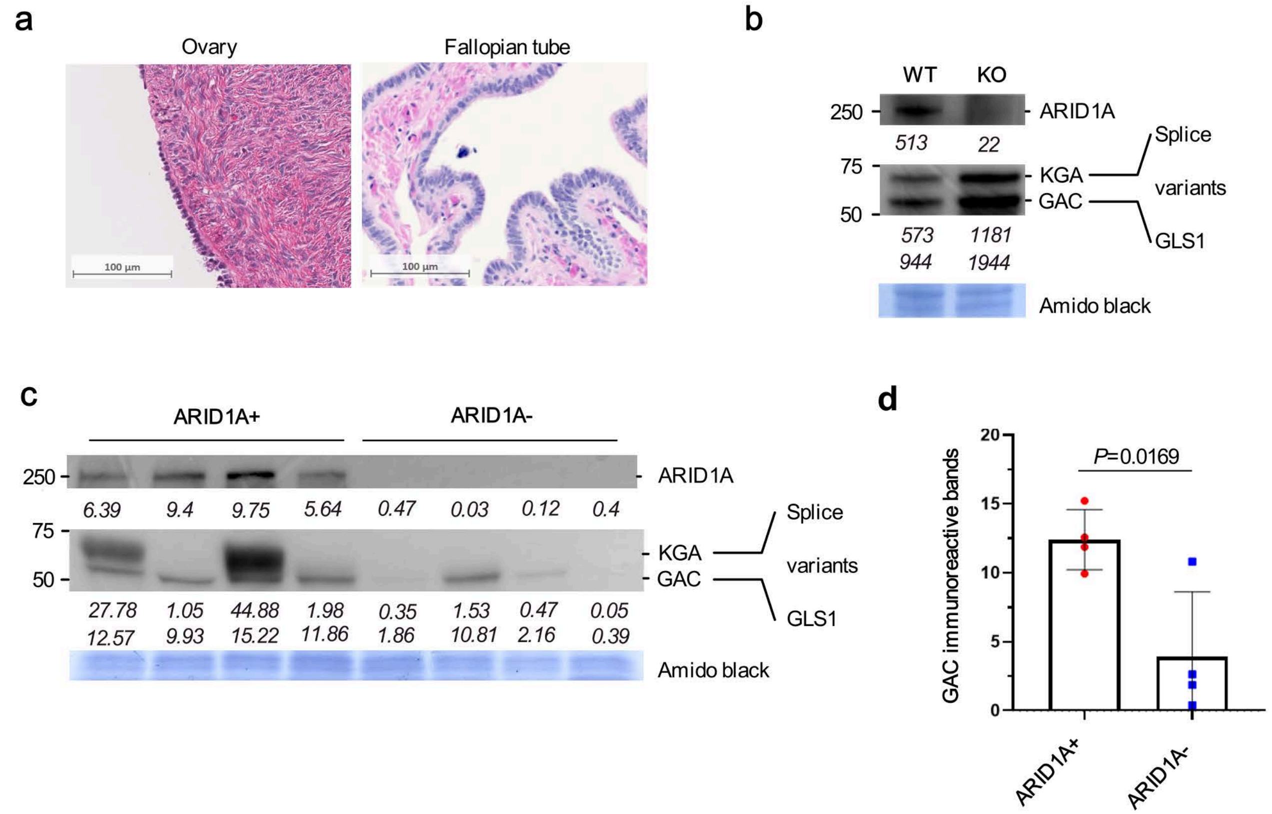

Supplementary Figure 1. a. H&E staining of ovary (*left*) and fallopian tube (*right*), corresponding to the GLS1 staining in Figure 4a. **b.** Western blot analysis of GLS1 levels in ARID1A KO and ARID1A WT RMG1 cells (*top*: ARID1A, *middle*: GLS1; *bottom*: amido black); the numbers represent the intensity of the above bands, normalized by amido black). **c.** GLS1 levels by Western blot in a cohort of representative cases of 4 ARID1A positive (*left*) and 4 ARID1A negative (*right*) OCCCs selected from our bigger cohort (*top*: ARID1A, *middle*: GLS1; *bottom*: amido black); the numbers represent the intensity of the above bands, normalized by amido black). **d.** graph representing the quantification of the lower band of GLS1 (GAC) in **c.**

Supplementary Table 1 Characteristics of ARID1A+ and ARID1A- patients. Numeric measures were tested using t-tests and categorical measures using Fisher's exact tests.

	Positive (N=36)	Negative (N=19)	Total (N=55)	p value
**Age**				0.792
Mean (SD)	59.8 (10.1)	60.5 (10.7)	60.0 (10.2)	
**BMI**				0.737
Mean (SD)	29.0 (7.4)	28.3 (8.3)	28.8 (7.6)	
**Endometriosis**				0.050
Yes	14 (38.9%)	13 (68.4%)	27 (49.1%)	
No	22 (61.1%)	6 (31.6%)	28 (50.9%)	
**Menopause**				1.000
N-Miss	3	1	4	
postmenopausal	24 (72.7%)	13 (72.2%)	37 (72.5%)	
premenopausal	9 (27.3%)	5 (27.8%)	14 (27.5%)	
**Ca125**				0.161
N-Miss	10	7	17	
Mean (SD)	808.8 (1159.7)	1757.4 (2957.1)	1108.4 (1925.7)	
**stage_4c**				0.067
I	15 (41.7%)	14 (73.7%)	29 (52.7%)	
II	9 (25.0%)	1 (5.3%)	10 (18.2%)	
III	11 (30.6%)	3 (15.8%)	14 (25.5%)	
IV	1 (2.8%)	1 (5.3%)	2 (3.6%)	
**stage_2c**				0.045
I	15 (41.7%)	14 (73.7%)	29 (52.7%)	
II/III/IV	21 (58.3%)	5 (26.3%)	26 (47.3%)	
**stage_bin**				0.533
I/II	24 (66.7%)	15 (78.9%)	39 (70.9%)	
III/IV	12 (33.3%)	4 (21.1%)	16 (29.1%)	

**Surgical outcome**				1.000
no residual disease	28 (77.8%)	16 (84.2%)	44 (80.0%)	
optimal < 1 cm	5 (13.9%)	2 (10.5%)	7 (12.7%)	
suboptimal >1 cm	3 (8.3%)	1 (5.3%)	4 (7.3%)	
**Chemotherapy response**				0.776
N-Miss	4	2	6	
complete	28 (87.5%)	14 (82.4%)	42 (85.7%)	
partial	2 (6.2%)	2 (11.8%)	4 (8.2%)	
stable	1 (3.1%)	0 (0.0%)	1 (2.0%)	
progressive	1 (3.1%)	1 (5.9%)	2 (4.1%)	
**Recurrence**				0.247
Yes	12 (33.3%)	10 (52.6%)	22 (40.0%)	
No	24 (66.7%)	9 (47.4%)	33 (60.0%)	
**Death by cancer**				0.047
N-Miss	0	1	1	
Yes	6 (16.7%)	8 (44.4%)	14 (25.9%)	
No	30 (83.3%)	10 (55.6%)	40 (74.1%)	
**Death**				0.071
Yes	8 (22.2%)	9 (47.4%)	17 (30.9%)	
No	28 (77.8%)	10 (52.6%)	38 (69.1%)	

Supplementary Table 2. Characteristics of patients divided by GLS1 expression levels (low: <100; intermediate: <200. ≥100; high: ≥200). Numeric measures were tested using ANOVA and categorical measures using Fisher's exact tests.

	< 100 (N=22)	100-200 (N=19)	> 200 (N=13)	Total (N=54)	p value
**Age**					0.441
Mean (SD)	61.8 (11.8)	57.8 (9.0)	60.3 (6.3)	60.0 (9.7)	
**BMI**					0.238
Mean (SD)	26.7 (8.5)	30.4 (7.3)	30.3 (6.2)	28.9 (7.7)	
**Endometriosis**					0.831
Yes	11 (50.0%)	8 (42.1%)	7 (53.8%)	26 (48.1%)	
No	11 (50.0%)	11 (57.9%)	6 (46.2%)	28 (51.9%)	
**Menopause**					0.765
N-Miss	0	1	3	4	
postmenopausal	15 (68.2%)	14 (77.8%)	8 (80.0%)	37 (74.0%)	
premenopausal	7 (31.8%)	4 (22.2%)	2 (20.0%)	13 (26.0%)	
**Ca125.5**					0.508
N-Miss	7	3	6	16	
Mean (SD)	1060.1 (1124.1)	1450.9 (2736.8)	421.0 (584.7)	1106.9 (1926.4)	
Median (Range)	550.0 (39.0, 3547.0)	241.4 (34.0, 10671.0)	194.0 (3.0, 1675.0)	370.0 (3.0, 10671.0)	
**stage_4c**					0.631
I	12 (54.5%)	8 (42.1%)	7 (53.8%)	27 (50.0%)	
II	3 (13.6%)	6 (31.6%)	1 (7.7%)	10 (18.5%)	
III	6 (27.3%)	4 (21.1%)	5 (38.5%)	15 (27.8%)	
IV	1 (4.5%)	1 (5.3%)	0 (0.0%)	2 (3.7%)	
**stage_2c**					0.780
I	12 (54.5%)	8 (42.1%)	7 (53.8%)	27 (50.0%)	
II/III/IV	10 (45.5%)	11 (57.9%)	6 (46.2%)	27 (50.0%)	
**stage_bin**					0.761

I/II	15 (68.2%)	14 (73.7%)	8 (61.5%)	37 (68.5%)	
III/IV	7 (31.8%)	5 (26.3%)	5 (38.5%)	17 (31.5%)	
**result_of_surgery**					0.954
no residual disease	18 (81.8%)	15 (78.9%)	11 (84.6%)	44 (81.5%)	
optimal < 1 cm	3 (13.6%)	2 (10.5%)	1 (7.7%)	6 (11.1%)	
suboptimal >1 cm	1 (4.5%)	2 (10.5%)	1 (7.7%)	4 (7.4%)	
**chemotherapy_response**					0.399
N-Miss	3	2	1	6	
complete	16 (84.2%)	12 (70.6%)	12 (100.0%)	40 (83.3%)	
partial	1 (5.3%)	3 (17.6%)	0 (0.0%)	4 (8.3%)	
stable	1 (5.3%)	0 (0.0%)	0 (0.0%)	1 (2.1%)	
progressive	1 (5.3%)	2 (11.8%)	0 (0.0%)	3 (6.2%)	
**recurrence_yn**					0.116
Yes	11 (50.0%)	9 (47.4%)	2 (15.4%)	22 (40.7%)	
No	11 (50.0%)	10 (52.6%)	11 (84.6%)	32 (59.3%)	
**death_by_cancer_yn**					0.040
N-Miss	1	0	1	2	
Yes	8 (38.1%)	6 (31.6%)	0 (0.0%)	14 (26.9%)	
No	13 (61.9%)	13 (68.4%)	12 (100.0%)	38 (73.1%)	
**death_yn**					0.200
Yes	10 (45.5%)	6 (31.6%)	2 (15.4%)	18 (33.3%)	
No	12 (54.5%)	13 (68.4%)	11 (84.6%)	36 (66.7%)	
